# Inhibition of Myelin-Cleaving Poteolytic Activities by Interferon-Beta in Rat Astrocyte Cultures. Comparative Analysis between Gelatinases and Calpain-II

**DOI:** 10.1371/journal.pone.0049656

**Published:** 2013-02-04

**Authors:** Tiziana Latronico, Maria Teresa Branà, Pasqua Gramegna, Anna Fasano, Gaetano Di Bari, Grazia Maria Liuzzi

**Affiliations:** Department of Biosciences, Biotechnology and Biopharmaceutics, University of Bari, Bari, Italy; Research Inst. of Environmental Med., Nagoya Univ., Japan

## Abstract

**Background:**

Proteolytic enzymes have been implicated in the pathogenesis of Multiple Sclerosis (MS) for both their ability to degrade myelin proteins and for their presence in MS plaques.In this study we investigated whether interferon-beta (IFN-β) could differently modulate the activity and the expression of proteolytic activities against myelin basic protein (MBP) present in lipopolysaccharide (LPS)-activated astrocytes.

**Methodology/Principal Findings:**

Rat astrocyte cultures were activated with LPS and simultaneously treated with different doses of IFN-β. To assess the presence of MBP-cleaving proteolytic activity, culture supernatants and cellular extracts collected from astrocytes were incubated with exogenous MBP. A MBP-degrading activity was found in both lysates and supernatants from LPS-activated astrocytes and was dose-dependently inhibited by IFN-β. The use of protease inhibitors as well as the zymographic analysis indicated the presence of calpain II (CANP-2) in cell lysates and gelatinases A (MMP-2) and B (MMP-9) in cell supernatants. RT-PCR revealed that the expression of CANP-2 as well as of MMP-2 and MMP-9 was increased in LPS-activated astrocytes and was dose-dependently inhibited by IFN-β treatment. The expression of calpastatin, the natural inhibitor of CANPs, was not affected by IFN-β treatment. By contrast, decreased expression of TIMP-1 and TIMP-2, the natural inhibitors of MMP-9 and MMP-2, respectively, was observed in IFN-β-treated astrocytes compared to LPS-treated cells. The ratio enzyme/inhibitor indicated that the effect of IFN-β treatment is more relevant to CANP-2 than on MMPs.

**Conclusions/ Significance:**

These results suggest that the neuroinflammatory damage during MS involves altered balance between multiple proteases and their inhibitors and indicate that IFN-β is effective in regulating different enzymatic systems involved in MS pathogenesis.

## Introduction

There is accumulating evidence that different classes of proteinases and their endogenous inhibitors can contribute to the pathogenesis of multiple sclerosis (MS), a chronic inflammatory disease of the central nervous system (CNS) characterized by breakdown of the blood brain barrier (BBB), infiltration of inflammatory cells into the CNS and demyelination [1]–[3].

Intracellular and extracellular proteolytic enzymes such as calpains and matrix metalloproteinases (MMPs) participate in demyelination, axon injury, apoptosis, and development of the inflammatory response including immune cell activation and extravasation, cytokine and chemokine activation/inactivation, complement activation and epitope spreading.

Calpains are a family of calcium-dependent cystein proteinases which degrade a wide variety of cytoskeletal, membrane associated and regulatory proteins. There are two major isoforms: calpain I (CANP-1) (µ-form) and calpain II (CANP-2 (m-form), which are ubiquously distributed and differ in their calcium requirement for activation [4].

Calpains are tightly regulated by the specific endogenous inhibitor calpastatin, which binds to the pro-enzyme. Calpains are upregulated by cytokines, and the presence of these proteinases in inflammatory cells suggests that their activation may be one of the several pathways leading to autoimmune demyelination in course of MS [5]. Different studies have indicated the involvment of calpains in the process of myelin degradation for both their ability to degrade myelin proteins such as myelin basic protein (MBP) and for their presence, at increased levels, in MS plaques of MS patients [2], [6], [7]. The source of increased calpain activity has been attributed to infiltrating inflammatory cells, activated microglia and reactive astrocytes [8], [9].

**Table:** **Table1.** Rat primer sequences used in RT-PCR.

Gene	Primer sequence	Product size (bp)
GAPDH	F: 5′-TCCCTCAAGATTGTCAGCAA-3′	308
	R: 5′- AGATCCACAACGGATACATT-3′	
MMP-2	F:5′-GTCACTCCGCTGCGCTTTTCTCG-3′	591
	R:5′-GACACATGGGGCACCTTCTGA-3′	
MMP-9	F:5′-CGGAGCACGGGGACGGGTATC-3′	541
	R:5′-AAGACGAAGGGGAAGACGCACATC-3′	
CANP-2	F:5′-CCCTCCCAACCTGTTCAAG-3′	440
	R:5′-GCCTCCAGTTCCCATCCA-3′	
TIMP-1	F:5′-CCAACCCACCCACAGACAGC-3′	493
	R:5′-GTGGCAGGCAGGCAAAGTGAT-3′	
TIMP-2	F:5′-CGTAGTGATCAGGGCCAAAGCAGT-3′	487
	R:5′-GTACCACGCGCAAGAACCATCAC-3′	
Calpastatin	F:5′-AGTAGTTCTGGACCCAATG­-3′	230
	R:5′-CCCCAGTAGACTTCTCTTTC-3′	

F: Forward primer; R: Reverse primer.

Despite calpains have been extensively studied over the past three decades, the pathophysiological roles of these enzymes remain poorly defined. In addition, although calpains have been indicated as target for treating acute neurodegenerative disorders such as brain ischemia [10], sparse data are available on the effect of MS therapy on calpain expression and activity.

By contrast, most of the studies performed in these last years focused on MMPs, extracellular enzymes involved in the remodelling of extracellular matrix (ECM), which have received great attention as mediators of inflammation and demyelination [11], [12]. Several reports have demonstrated the increase of gelatinase B (MMP-9) in brain tissue, cerebrospinal fluid (CSF) and blood circulation of patients with relapsing-remitting (RR)-MS [13]–[16] suggesting that this enzyme can contribute to BBB opening and demyelination. The involvement of MMP, and in particular of MMP-9, in demyelination is also suggested by the ability of this enzyme to degrade MBP *in vivo* and *in vitro* [17]–[19]. It has been demonstrated that the treatment of MS patients with interferon-beta (IFN-β) reduces MMP-9 serum levels and restores the integrity of the barrier [20], [21]. Proteolysis by MMPs is balanced by endogenous tissue inhibitors of MMPs (TIMPs), which bind to MMPs forming a complex which inactivates the enzyme. Serum and CSF levels of TIMP-1 decrease in RR-MS patients [15], while IFN-β treatment increases serum levels of TIMP-1 in RR-MS patients [22].

On the basis of these considerations in the present paper we investigated the presence of MBP-cleaving proteolytic activity in lipopolysaccharide (LPS)-activated astrocytes and demonstrated the increase of different proteolytic activities in cell lysates and supernatants and their inhibition by IFN-β. By using zymography we identified CANP-2 as the main proteolytic activity present in cell lysates, while only gelatinases A (MMP-2) and B were released in cell culture supernatants. We also studied whether IFN-β could differently modulate the expression of CANP-2, MMP-2 and MMP-9 in relation to their natural tissue inhibitors in LPS-activated astrocytes, demonstrating a higher responsiveness of the calpain/calpastatin system to IFN-β in comparison with the MMP/TIMP system.

## Materials and Methods

### Reagents

Dulbecco's modified Eagle's medium (DMEM), fetal bovine serum (FBS), penicillin and streptomycin, Trypsin/EDTA and TAE buffer were obtained from GIBCO (Paisley, Scotland). Gelatin, DNase 1, poly-L-lysine (PLL), trypsin, lipopolysaccharide (LPS), Trypan Blue, bovine serum albumin (BSA), casein, 1,10 phenanthroline (PA), phenilmethylsulfonyl fluoride (PMSF), iodacetic acid (IA), E-64, EDTA, were provided by Sigma (St. Louis, MO, USA.). IFN-β was from R&D Systems (Minneapolis, MN) Standard proteins and R-250 Coomassie Brilliant Blue were purchased from Bio-Rad (Hercules, CA, USA).

Anti glial fibrillary acidic protein (GFAP) antibodies and calpain II standard were purchased from Calbiochem (La Jolla, CA). Purified MMP-2 and MMP-9 were purchased from Alexis Biochemicals (San Diego, CA, USA). The MMP-9 was derived from human neutrophil granulocytes and thus also contained covalent MMP-9-NGAL complex. Primer pairs specific for MMP-2, MMP-9. calpain-II, TIMP-1, TIMP-2, calpastatin and GAPDH were from Sigma Genosys (Cambridge, UK). RNeasy mini kit was from Qiagen (Valencia, CA, USA). All the reagents for the RT-PCR were from Invitrogen (San Diego, CA).

### Ethics Statement

All experimental procedures involving animals were carried out in strict accordance with the recommendations in the NIH Guide for the Care and Use of Laboratory Animals and approved by the Institutional Animal Care and Use Committee of University of Bari, Italy (Permit Number: 23–98-A). All efforts were made to minimize the number of animals used and to ameliorate their suffering.

### Preparation of astrocyte cultures

Astrocytes were prepared from primary cell cultures of neocortical tissues from 1-day-old rats as described by Nakajima et al.[23]. Briefly, brains were cleaned of meninges and blood vessels and the dissected neocortical tissues were minced by passage through a stainless steel mesh (40 mesh) and incubated with 0.25% trypsin and 0.01% DNase in DMEM for 10 min at 37°C. After addition of fetal bovine serum (FBS), the dissociated cells were passed through a 100 mesh and viability of cells was assessed by Trypan Blue dye exclusion. Cells were plated in poly-L-lysine (PLL)-coated flasks (75 cm^2^) at a density of 1.5×10^7^ viable cells/flask in DMEM, 100 U/ml penicillin/100 µg/ml streptomycin, 10% FBS and maintained at 37°C in a 5% CO_2_ incubator with a renewal of medium twice a week. After 7–10 days in culture, microglia and oligodendrocytes were separated from astrocytes by mechanical dislodging and then the astrocytes were obtained by trypsinization with 0.25% trypsin/0.02% EDTA) [24]. Astrocytes were purified by three repetitions of replating and trypsinization to deplete cultures of microglia and oligodendrocytes. The purity of the final cell culture was assessed by immuno-staining for glial fibrillary acidic protein (GFAP) using a polyclonal anti-GFAP antibody. More than 98% of the cells were GFAP-positive in all the preparations.

### Treatment of astrocytes with IFN-β

Confluent cultures of astrocytes, plated in 96-well-plates, were washed twice with serum-free DMEM, activated with LPS (10 µg/ml) and simultaneously treated with a recombinant rat IFN-β at the final concentrations of 50, 100 and 500 U/ml. The incubations were performed for 24 h at 37°C in 100 µl of serum-free DMEM. At the end of the incubation period the culture medium was collected and cells were lysed with a buffer containing 50 mM Hepes, pH 7.6, 150 mM NaCl, 1% Triton X-100, 5 mM EDTA, 5 mM EGTA, 10 mM 2-mercaptoethanol, 0.1 mM phenilmethylsulfonyl fluoride (all from Sigma). After centrifugation to remove particulate debris, lysates and cell culture supernatants were stored at −80°C until use. Cells from other wells were subjected to total RNA extraction for RT-PCR analysis. Controls consisted of lysates and supernatants from unstimulated and untreated astrocytes (negative control) and LPS-activated cells (positive control).

### Detection of MBP-cleaving proteolytic activity

For the assessment of MBP-cleaving proteolytic activity, 10 µg of bovine MBP, prepared according to Deibler et al. [25] were incubated with different amounts of cell culture supernatant or lysate and brought to a final volume of 100 µl with 50 mM Tris-HCl (pH 7.5). The reaction was initiated by the addition of the substrate MBP. After incubation at 37°C for 1–24 h the reaction was stopped by the addition of 1 ml of cold acetone (Sigma). After 1 h incubation at −20°C, samples were centrifuged, resuspended in 10 µl of loading buffer and subjected to SDS-PAGE in 15% acrylamide thick slab gels. After setting the right conditions, all the experiments were performed by incubating 10 µg of MBP with 30 µl of cell lysate or 50 µl of supernatant for 24 h at 37°C. In each test, as controls, MBP was incubated without culture supernatant or lysate (MBP control) or with culture supernatant or lysate from unstimulated astrocytes (CTRL). In another set of experiments cell culture supernatants or lysates from LPS-activated astrocytes were incubated with 10 µg of bovine serum albumin (BSA) in the same experimental conditions used for the incubation with MBP.

To investigate the nature of the proteolytic activity on MBP, 30 µl of cell lysate or 50 µl of supernatant from LPS-activated astrocytes were incubated with 10 µg of MBP in the presence of different proteinase inhibitors. Proteins stained with Coomassie brilliant blue R-250 were quantified, after scanning densitometry of gels, by using the IMAGE Master 1D program (Pharmacia Biotech, Uppsala, Sweden) and proteinase activity was expressed as percentage of not degraded MBP compared to MBP control.

### Separation and analysis of CANP-2 activity

Calpain activity in cell culture supernatants and lysates was detected by casein zymography using a protocol described by Croall et al. [26]. Briefly, casein (0.2%, w/v) was copolimerized with 12% (w/v) acrylamide, 0.32% (w/v) bisacrylamide, 35 mM Hepes and 43 mM imidazole (pH 7.4) and poured into minigel casts. A pH 7.4 buffer composed of 35 mM Hepes and 43 mM imidazole, as described by McLellan [27], was used for a continuos buffer system within the gel and for electrophoresis. Thirty µl of lysates or culture supernatant corresponding to about 15 µg and 3 µg of proteins, respectively, were supplemented with 5 µl of sample buffer (150 mM Tris-HCl (pH 6.8), 30% glycerol, 2 mM 2-mercaptoethanol, 0.004% (w/v) bromophenol blue) and were run on nondenaturing conditions. Electrophoresis was performed at 4°C with a 45 min pre-run at 125 V and then at 125 V for approximately 3 h. After electrophoresis, calpain activity was developed by incubating the gels in 20 mM Tris-HCl (pH 7.4), 10 mM dithyothreitol (DTT), 4 mM CaCl_2_ at room temperature with slow shaking for 60 min (with two changes of developing buffer), followed by an overnight incubation (14–18 h) in the same buffer at room temperature. Gels were then stained with Coomassie Brillant Blue R-250 and destained in methanol/acetic acid/water. Calpain activity was detected as a white band on a blue background and was identified by colocalization on the zymogram with standard CANP-2.

For the quantitation of calpain levels, gels were subjected to scanning densitometry and image analysis using the IMAGE Master 1D program (Pharmacia Biotech, Uppsala, Sweden).

CANP-2 levels were expressed as optical density (OD) × mm^2^, representing the scanning area under the curves which takes into account both brightness and width of the substrate lysis zone.

### Detection of gelatinases by zymography

Gelatinases in cell culture supernatants were detected by gelatin-zymography as described in Liuzzi et al. [28].

Briefly, 50 µl of culture supernatant was supplemented with 30 µl of electrophoresis loading buffer containing SDS. Samples were then separated in a 7.5% polyacrylamide gel copolymerized with 0.1% (w/v) gelatin. Stacking gels contained 5.4% polyacrylamide. Electrophoresis was performed at 4°C for approximately 18 h at 80 V. After electrophoresis, gels were washed for 2×20 min in 2.5% (w/v) Triton X-100/ 10 mM CaCl_2_ in 50 mM Tris-HCl, pH 7.4 (washing buffer) in order to remove SDS, then incubated for 24 h at 37°C in 1% (w/v) Triton X-100/ 50 mM Tris-HCl/ 10 mM CaCl_2_, pH 7.4 (developing buffer).

For the development of enzyme activity, the gels were stained with Coomassie brilliant blue R-250 and destained in methanol/acetic acid/H_2_O. Gelatinase activity was detected by densitometrical analysis of gels as described above.

### Total RNA isolation and reverse transcriptase-polymerase chain reaction (RT-PCR)

Total cellular RNA was isolated from astrocytes using an RNeasy mini kit according to the manufacturer's instructions. RNA was ethanol-precipitated, resuspended in RNase-free water and its quantity and purity was determined spectrophotometrically at 260–280 nm. RNA samples were stored at −70°C until use. Heat-denatured RNA (5–10 µg) was used for cDNA synthesis in a total volume of 20 µl at 42°C for 50 min using 5x first strand synthesis buffer, 0.5 mM of each deoxynucleotide triphosphate (dNTP), 500 ng of random primers, 10 mM DTT and 200 units of reverse transcriptase. The cDNA (5 µl) was used in each reaction for amplification of one message at a time by the polymerase chain reaction (PCR) with rat primers specifically designed for the genes of interest (Table 1). For specific amplification, Taq DNA Polymerase was used in all RT-PCR experiments performed in a thermal cycler (PTC-100 Programmable Thermac Controller, MJ Research, Inc) using the following program: one cycle of denaturation at 94°C for 4 min; 25 cycles including template denaturation at 94°C for 1 min, template-primer annealing for 1 min, at 59°C for MMP-2, MMP-9 and GAPDH, at 57°C for TIMP-1, at 60°C for TIMP-2 and 50°C for CANP-2 and calpastatin, and primer extension at 72°C for 1 min; one cycle of extension of all nascent DNA at 72°C for 6 min. The RT-PCR product (10 µl) from each reaction tube was resolved on a 1.5% agarose gel by electrophoresis at 2 V/cm in TAE buffer, pH 8.3 buffer and visualized by ethidium bromide staining. Gels were then photographed and processed for densitometric analysis as described for protein gels. Standard molecular size markers, negative control (PCR mix without sample cDNA) and positive controls were included in each PCR assay.

The mRNA expression of each target gene was normalized to that of glyceraldehyde 3-phosphate dehydrogenase (GAPDH), which served as an internal control.

### Statistical analysis

Data from at least three different experiments were used for statistical analysis. Values were compared using one-way analysis of variance (ANOVA) followed by the post-hoc Student Newman-Keuls test for multiple comparisons. Statistical significance was considered relevant for p values <0.05. Data were analyzed by the GraphPad Prism 5.0 (GraphPad Software, San Diego, CA).

## Results

### Degradation of purified MBP by proteolytic activities present in astrocyte lysates and supernatants

**Figure 1:**
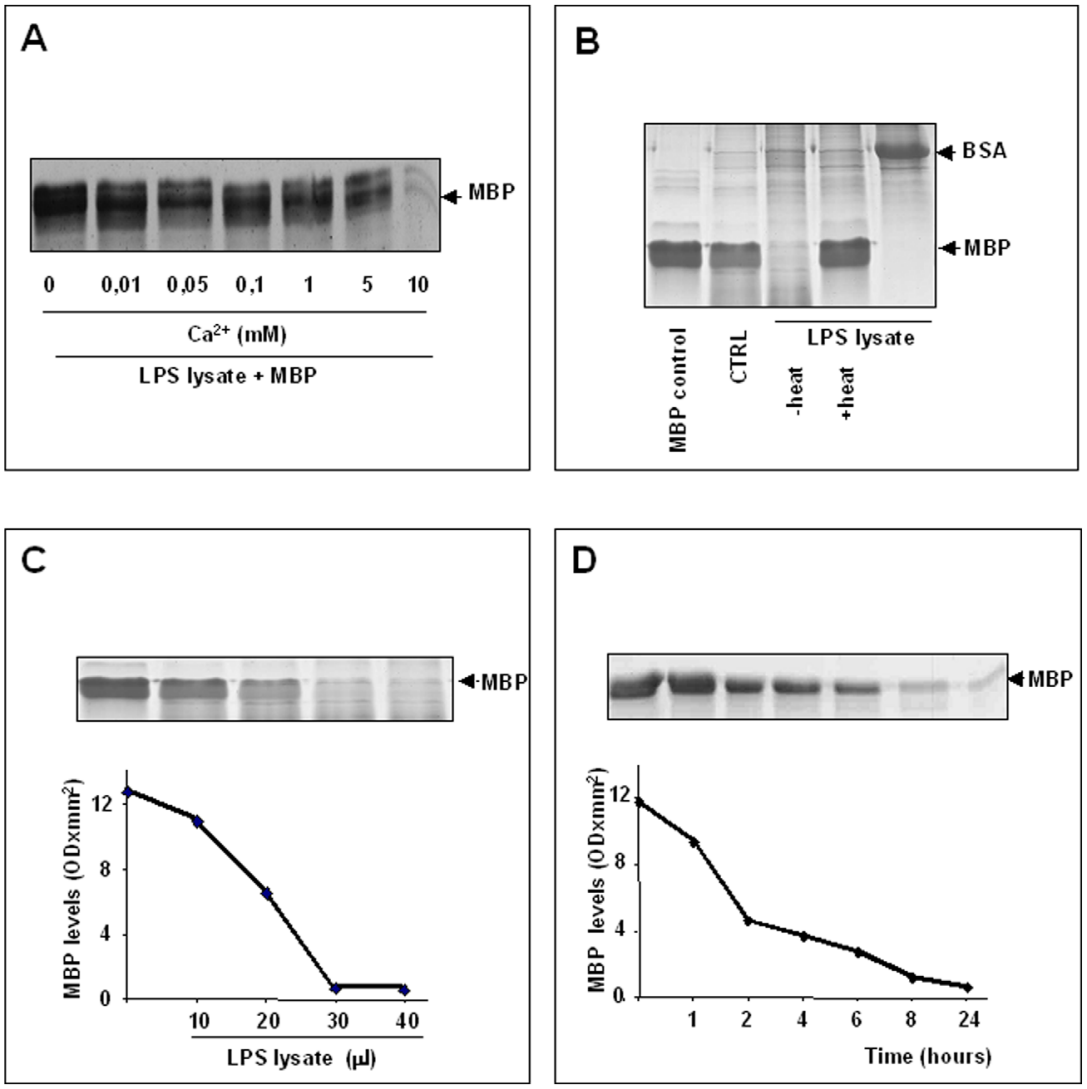
Degradation of MBP by proteolytic activities present in astrocyte lysates. Lysates from LPS-activated astrocytes were incubated with exogenous MBP, then subjected to SDS-PAGE as described in text. As controls, MBP was incubated without cell lysate (MBP control) or with lysate from unactivated astrocytes (CTRL). A, Calcium dependency of MBP degradation. B, Substrate specificity of proteolytic activity present in LPS-activated lysates towards MBP. C, Dose-dependent degradation of MBP by LPS-activated lysates from astrocytes after incubation at 37°C for 24 h. D, Time-course of the MBP degradation by 30 μl of LPS-activated lysate. In the lower part of C and D MBP degradation is expressed as optical density (OD × mm^2^) as calculated by densitometrical analysis of gel.

Astrocyte lysates and supernatants were tested for the presence of MBP-degrading proteolytic activity. As shown in Figure 1, degradation of MBP was observed when this protein was incubated with lysates from LPS-activated astrocytes in the presence of increasing concentrations of calcium (A). The degradation of MBP yielded fragments which were identified as MBP degradation products by immunoblotting analysis using a monoclonal anti-MBP antibody (data not shown). No degradation of MBP was observed when the protein was incubated with the same lysate from LPS-activated astrocytes after heat inactivation for 10 min at 100°C or with the lysate from non-activated control cells (CTRL) (B). As shown in Figure 1B the proteolytic activity present in LPS-activated astrocytes did not degrade BSA, indicating a substrate specificity towards MBP. The degradation of MBP significantly increased with increasing amounts of lysate (C) and was also dependent on the reaction time (D). These results indicate that LPS-activation of astrocytes induces MBP-cleaving, calcium-dependent, heat-sensitive proteolytic activities. Similar results were found when MBP was incubated with cell culture supernatants from LPS-activated astrocytes (data not shown).

### Characterization of MBP-cleaving proteolytic activities

**Figure 2:**
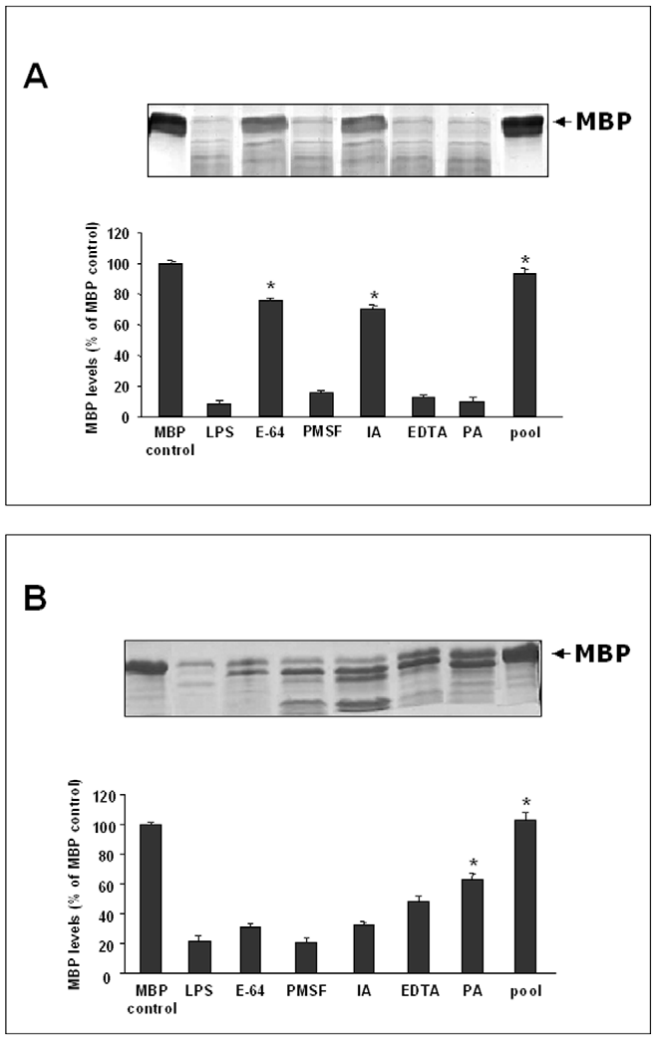
Effect of proteinase inhibitors on the degradation of MBP by lysates or supernatants. 30 µl of lysates (A) or 50 µl of supernatants (B) from LPS-activated astrocytes were incubated for 24 h at 37°C with 10 µg of exogenous MBP in the presence of CaCl_2_ (10 mM) and: 20 µM E-64; 2 mM PMSF; 10 mM IA; 2 mM EDTA; 4 mM PA or a pool containing all the inhibitors at the used concentrations. The positive control (LPS) is represented by MBP incubated with lysates or supernatants from LPS-activated astrocytes in the absence of inhibitors. Histograms show the percentage of non-degraded MBP compared to negative control (MBP control), represented by MBP incubated in the same experimental conditions without cell culture supernatant or lysate. Results represent the mean ± SD of three separate experiments using lysates or supernatants from different cell populations. Statistically significant inhibition of MBP degradation in comparison to that of positive control is indicated with * (One way ANOVA followed by Student – Newman – Keuls post hoc test; * =  p<0.05).

In order to better characterize the MBP-degrading activities, lysates and supernatants from LPS-activated astrocytes were incubated with MBP in the presence of various protease inhibitors specific for different types of proteinases. As shown in Figure 2 none of the inhibitors, used alone, was effective in completely blocking the MBP degradation in both cell lysates (A) and supernatants (B), whereas MBP degradation was blocked when a pool of proteinase inhibitors was used, indicating that cell lysates and supernatants from LPS-activated astrocytes contained different types of myelin-cleaving proteolytic activities. Degradation of MBP in cell lysates was largely due to cystein proteinases, as shown by the reduction of MBP degradation by the cystein proteinase inhibitors E-64 and iodoacetic acid (IA). Indeed, these inhibitors substantially inhibited MBP degradation of 80% and 79% respectively.

By contrast, PMSF, a serine proteinase inhibitor, EDTA and 1,10 phenantrolin (PA), two metalloproteinase inhibitors, were no effective in inhibiting MBP degradation in cell lysates. On the contrary, in cell supernatants EDTA and PA inhibited MBP degradation of 50% and 65% respectively, indicating that MMPs were the dominant contributors to MBP degradation in cell culture supernatants.

### Inhibition of myelin-cleaving proteolytic activities by IFN-β

**Figure 3:**
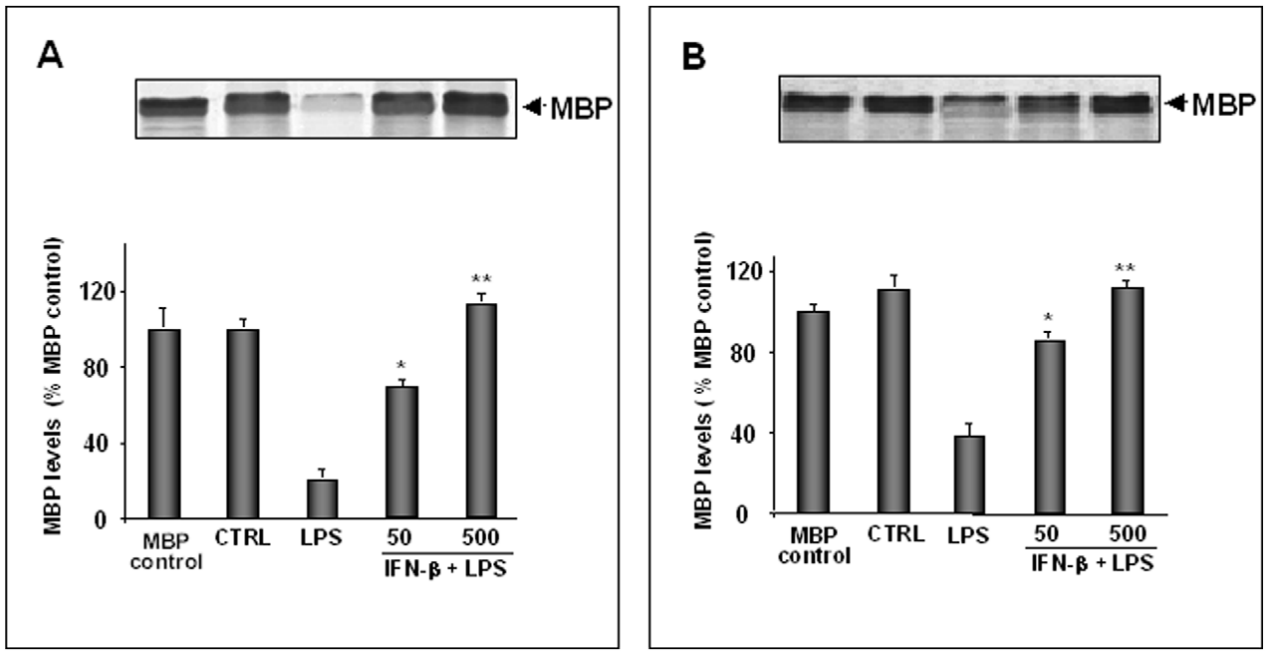
Effect of IFN-β on MBP-cleaving proteolytic activities. Primary astrocytes (1×10^5^ cells/ml), incubated in serum-free DMEM, were treated with IFN-β at the indicated concentrations (U/ml) in the presence of LPS (10 µg/ml). After 24 h of incubation culture supernatants were collected and cells were lysed. 30 µl of lysates (A) or 50 µl of supernatants (B) were incubated for 24 h at 37°C with 10 µg of exogenous MBP in the presence of CaCl_2_ (10 mM) and then subjected to SDS-PAGE. As controls, MBP was incubated without supernatants or lysates (MBP control) or with supernatants or lysates from non-activated astrocytes (CTRL). The positive control was represented by supernatants or lysates from LPS-activated astrocytes (LPS). Histograms show the percentage of non-degraded MBP compared to MBP control. Results represent the mean values ± SD of three separate experiments using lysates or supernatants from different cell populations. Statistically significant inhibition of MBP degradation in comparison to that of positive control is indicated with * (One way ANOVA followed by Student – Newman – Keuls post hoc test; * =  p<0.05; **P<0.005).

To test the effect of IFN-β on MBP-cleaving proteolytic activities present in LPS-activated lysates and supernatants, in another set of experiments astrocytes were activated with LPS and simultaneously treated with increasing doses of IFN-β. As shown in Figure 3, IFN-β treatment induced a dose-dependent inhibition of MBP degradation in both lysates (A) and supernatants (B). The inhibitory effect of IFN-β was comparable to that obtained by using the pool of proteinase inhibitors, as shown in Figure 2.

### Detection of CANP-2, MMP-2 and MMP-9 levels by zimographic analysis and inhibitory effect of IFN-β

**Figure 4:**
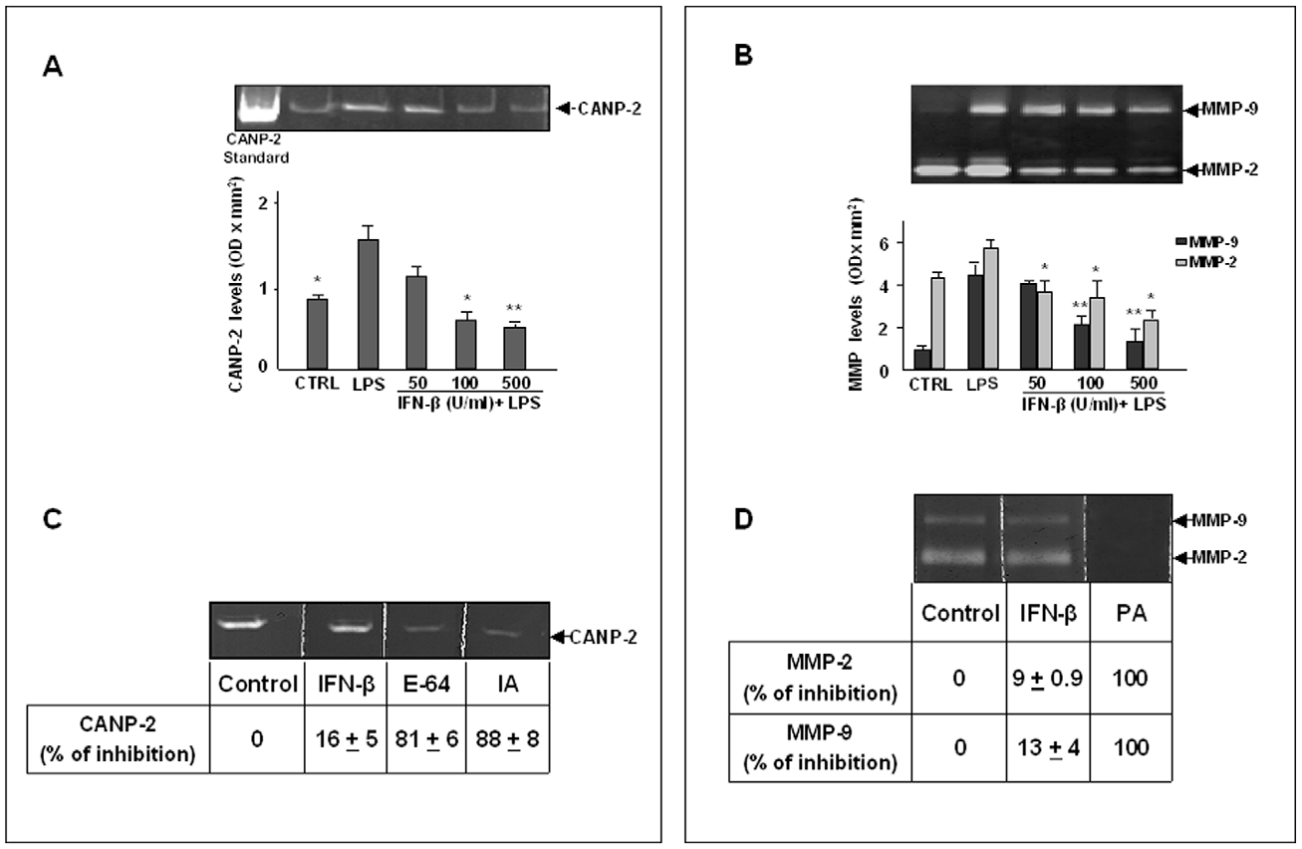
Effect of IFN-β treatment on CANP-2, MMP-2 and MMP-9 levels and activity. Primary astrocytes (1×10^5^ cells/ml), incubated in serum-free DMEM, were treated with IFN-β at the indicated concentrations (U/ml) in the presence of LPS (10 µg/ml). After 24 h of incubation cell lysates and supernatants were prepared and subjected to casein- (A) or gelatin-zymography (B). The negative control (CTRL) was represented by non-activated and untreated cells. CANP-2 present in lysates was identified by comigration with 0.5 µg of CANP-2 standard. MMP-2 and MMP-9 were identified by their apparent molecular mass of 67 and 92 kDa, respectively, using pre-stained molecular weight markers (Bio Rad). Histograms show the quantitation of CANP-2, MMP-2 and MMP-9 levels, expressed as optical density (OD) × mm^2^, calculated after scanning densitometry and computerized analysis of gels. Results represent the mean ± SD of three separate experiments performed with different cell populations. Statistically significant values different from positive control, represented by lysates or supernatants from LPS-activated astrocytes (LPS), are indicated with * (One-way Anova followed by Student – Newman – Keuls post hoc test; *P<0.05; **P<0.005). The effect of IFN-β on CANP-2 or MMP-2 and MMP-9 activity was also evaluated in a cell-free system. Purified standard CANP-2 (0.5 µg) was subjected to casein-zymography (C). Purified standard MMP-2 (0.35 µg) and MMP-9 (0.88 µg) were subjected to gelatin-zymography. After the SDS-electrophoretic run, gels were cut in slices and each slice was incubated overnight, at room temperature, in the incubation medium (20 mM Tris-HCl, 10 mM DTT, 4 mM CaCl_2_, pH 7.4) in the absence (Control) or in the presence of IFN-β (500 U/ml). E-64 (20 µM), IA (10 mM) or PA (4 mM) were used as positive controls. Staining and destaining of the gels revealed that IFN-β was not able to inhibit the activity of CANP-2 as well as MMP-2 and MMP-9 as already observed for E-64 and IA or PA. The percentages of inhibition, calculated in comparison to control, are reported in the lower part of Figure 4C and D.

Zymographic analysis on casein- or gelatin-copolymerized gels was performed to further characterize the MBP-cleaving proteolytic activities present in astrocyte supernatants and lysates and to study the effect of IFN-β. As reported in Figure 4A, in astrocyte lysates a band of digestion of approximately 100 kDa was observed on gels, identified as CANP-2 by co-migration with a standard CANP-2. CANP-2 levels were significantly increased in LPS-activated lysates in comparison to unstimulated lysates (CTRL). By contrast, the treatment with IFN-β induced a dose-dependent inhibition of CANP-2 levels in lysates from LPS-activated astrocytes. No bands of digestion were detected when cell culture supernatants were analyzed on casein-copolymerized gels (data not shown). As reported in Figure 4B, the zymographic analysis on gelatin-copolymerized gels indicated that the main proteolytic activities present in cell supernatants were represented by MMP-2 and MMP-9, which were also dose-dependently inhibited by IFN-β. MMP-2 and MMP-9 were not detectable in astrocyte lysates as observed by the zymographic analysis using gelatin-copolimerized gels (data not shown). Results from multiple experiments with different cell populations are reported in the histograms.

We also tested the ability of IFN-β to inhibit CANP-2, MMP-2 and MMP-9 activity by using an *in vitro* assay. For these experiments, aliquots of a standard preparation of CANP-2 were separated by casein-zymography (C) while standard preparations of MMP-2 and MMP-9 were separated by gelatin-zymography (D). After the run, the zymograms were cut in lanes and the lanes were incubated in developing buffer in the absence (control) or in the presence of IFN-β at the final concentration of 500 U/ml. As positive control, the casein zymograms were incubated in the presence of 20 µM E-64 or 10 mM IA, while the gelatin zymograms were incubated in the presence of PA. As shown in Figure 4C and 4D, IFN- β did not exert any direct inhibition on the enzymatic activity of CANP-2, MMP-2 and MMP-9. By contrast, E-64 and IA, two inhibitors of CANP-2 which were able to substantially inhibit MBP degradation by astrocyte lysates, partially blocked the activity of CANP-2 (C), whereas PA, a specific inhibitor of MMPs, completely inhibited the activity of both MMP-2 and MMP-9 (D). The percentages of *in vitro* inhibition of CANP-2, MMP-2 and MMP-9 activity in comparison to control are reported in the lower part of Fig. 4C and 4D.

### Effect of IFN-β on mRNA expression of MMP-2/TIMP-2, MMP-9/TIMP-1, CANP-2/calpastatin in LPS-activated astrocytes

**Figure 5:**
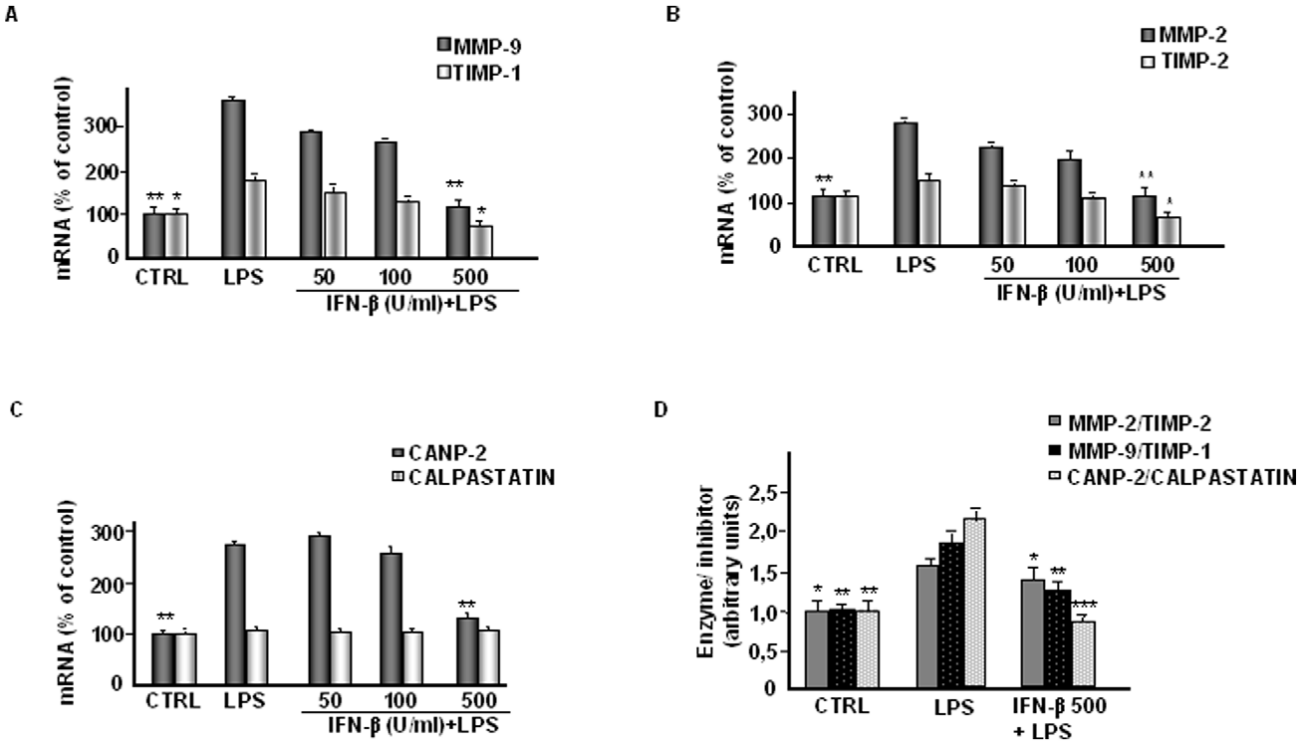
Effect of IFN-β on mRNA expression of the systems enzyme/inhibitor in astrocytes. Primary astrocytes (1×10^5^ cells/ml), incubated in serum-free DMEM, were treated with IFN-β at the indicated concentrations (U/ml) in the presence of LPS (10 µg/ml). The control (CTRL) was represented by non-activated and untreated cells. After 24 h of incubation total cellular RNA was isolated as described in the Methods section and analyzed by RT-PCR, using the primer pairs specific for MMP-2, MMP-9, TIMP-2, TIMP-1, CANP-2, calpastatin and GAPDH. Histograms in A-C, represent the quantitation of target genes, after normalization with GAPDH, from three separate experiments with different cell populations. CTRL mRNA levels were set at 100% and the LPS or IFN-β treatments were represented as percent of control (mean±SD). Values statistically different from positive control (LPS-activated cells) are represented by * (One-way Anova followed by Student – Newman – Keuls post hoc test; *P<0.05; **P<0.005). D, Effect of IFN-β on MMP-9/TIMP-1, MMP-2/TIMP-2 and CANP-2/calpastatin mRNA ratios. The MMP-9/TIMP-1, MMP-2/TIMP-2 and CANP-2/calpastatin mRNA ratios were calculated from the levels of individual genes, expressed as percent of negative control. The histogram represents the mean values ± SD of three separate experiments performed with different cell populations. Statistically significant values different from positive control (LPS-activated astrocytes) are indicated with * (One-way Anova followed by Student – Newman – Keuls post hoc test; *P<0.05; **P<0.005; ***P<0.001).

We also evaluated the effect of IFN-β on the mRNA expression of CANP-2 and its natural inhibitor calpastatin as well as of MMP-9 and MMP-2 in relation to their natural inhibitors TIMP-1 and TIMP-2, respectively. RT-PCR analysis indicated that LPS significantly induced the expression of MMP-2, MMP-9 as well as of TIMP-1. The treatment with IFN-β dose-dependently inhibited the expression of both MMP-9 and MMP-2, as well as of TIMP-1 and TIMP-2 in LPS-treated astrocytes (Figure 5A–B). A distinct expression profile was observed for the system CANP-2/calpastatin. In fact, LPS was able to up-regulate CANP-2 mRNA but was ineffective on calpastatin mRNA. Similarly, while CANP-2 expression was dose-dependently inhibited by IFN-β in LPS-activated astrocytes, the expression of calpastatin was not affected by IFN-β treatment (Figure 5C).

We also calculated the ratio of enzyme/inhibitor mRNA expression which provides a more comprehensive way to look at the global proteolytic balance. As shown in Figure 5D in LPS-activated cells we observed a statistically significant increase of the ratio enzyme/inhibitor mRNA expression that is more pronounced for the CANP-2/calpastatin system than for the MMP/TIMP systems. By contrast, since calpastatin expression was not regulated, the inhibitory effect of IFN-β treatment was more relevant on CANP-2 than on MMPs.

## Discussion

Multiple sclerosis is an autoimmune disease characterized by the invasion of reactive T cells into the CNS. Once in the brain parenchyma, these cells activate the resident cells such as astrocytes and microglia, inducing them to release myelinotoxic factors [29]–[31]. The activation of glial cells leads to the production of intracellular and extracellular proteinases that may be responsible for the degradation of the proteins of the myelin sheath. Given the importance of myelin basic protein (MBP) in the compaction of myelin, its release from the sheath and the degradation within the CNS may have relevant importance in the pathogenesis of MS. Therefore, the identification of the proteinases responsible for myelin damage could be useful to find new therapeutic strategies in demyelinating diseases such as MS. The block of these proteinases may contribute to the clinical improvements of MS patients. In this study, by using an experimental model successfully utilized in other studies [28], [32], [33] we have investigated whether the activation of astrocytes with LPS may induce the production of MBP-cleaving proteolytic activities in culture supernatants and cell lysates.

Our results indicate that LPS-activated astrocytes produce both intracellular and extracellular proteolytic activities that are calcium-dependent, heat-sensitive and specific for MBP. The use of specific proteinase inhibitors suggested that the nature of these proteinases was different. Indeed, the predominant extracellular proteolytic activities released in cell supernatants were represented by metalloproteinases (MMPs) as suggested by the finding that PA and EDTA, two inhibitors of metalloproteinases, are the most effective in blocking MBP degradation by cell supernatants. In addition, the incubation of cell supernatants with MBP produces MBP-specific degradation fragments which could correspond to the “remnant epitopes” of MBP after digestion with MMP-9, already described by other authors [34]. Furthermore, in our laboratory we have already demonstrated that the activation of both astrocytes and microglia by LPS induces the release of MMP-2 and MMP-9 and their inhibition by IFN-β [28]. Intracellular proteinases present in cell lysates were inhibited by inhibitors of cysteine proteinases suggesting the presence of calpains. This hypothesis was confirmed by the casein-zymographic analysis of cell lysates which showed the presence of CANP-2 that was induced by LPS and inhibited by IFN-β. In this respect, it should be noted that despite the therapeutic impact of IFN-β in reducing clinical relapses and MRI activity and the recognized role of calpains in the pathogenesis of MS, the information on the potential of IFN-β in modulating calpains are limited. Recent studies have suggested that the inhibition of calpains by synthetic inhibitors is effective in attenuating clinical symptomps of experimental allergic encephalomyelitis (EAE) by reducing inflammation, axonal damage and neuronal loss [4]–[35], [36]. Our study, therefore, expands these investigations demonstrating that IFN-β, the main drug used for the treatment of MS patients, exerts inhibitory activity on calpains.

One interesting result of this study is represented by the experimental evidence that IFN-β was able to completely block the degradation of MBP in both supernatants and cell lysates with the same efficiency of a cocktail of inhibitors of the different classes of proteinases indicating that IFN-β as single drug may be able to inhibit different proteolytic enzymes active towards myelin.

Both calpains and MMPs are enzymes involved in the pathogenesis of MS. Their involvement in mechanisms of BBB disruption and myelin degradation are supported by different experimental evidences. Therefore, blocking these proteinases may inhibit multiple pathways linked to disability.

The balance between enzymes and their inhibitors is an important regulatory factor *in vivo* since it determines the net activity of the enzymes and regulates their action in both physiological and pathological conditions. The activity of both calpains and MMPs is regulated at different levels. Posttranscriptional regulation of MMPs includes their secretion as latent enzymes and proenzyme activation in the extracellular milieu by different proteinases. Inhibition of MMP activation and proteolytic activity in the extracellular milieu is controlled by a unique family of natural tissue inhibitors (TIMPs) which form with active MMPs stable, non-covalent enzyme-inhibitor complexes [37]. Similarly, calpains are regulated *in vivo* by their endogenous inhibitor calpastatin, which binds to the pro-enzyme preventing its activation.

Therefore, it is important to determine the effect of IFN-β not only on the enzymes but rather on the system enzyme/inhibitor, in order to evaluate its effect on the enzyme that is free from the binding with the inhibitor and therefore active. In this respect, in a previous paper it has already been shown in other cell types that IFN-beta modulates the protein level ratios of MMP/TIMP [38].

In this paper we demonstrated that IFN-β inhibited in a dose-dependent manner the mRNA expression of MMP-9 and MMP-2, as well as of their natural inhibitors TIMP-1 and TIMP-2. Differently, IFN-β dose-dependently inhibited CANP-2 mRNA, but had no effect on calpastatin mRNA. Therefore, following IFN-β treatment, the ratio CANP-2 mRNA/calpastatin mRNA tends to decrease more than the ratio MMP mRNA/TIMP mRNA. Considering the importance that the ratio CANP/calpastatin plays in regulating CANP activation and the contribution of calpains to the development of MS, this result is extremely important since it may suggest that IFN-β may also act by counteracting the increase of CANP/calpastatin ratio. Hovewer, the significant reduction of MMP-2/TIMP-2 and MMP-9/TIMP-1 ratios suggests that the inhibitory effects of IFN-β on MMP-2 and MMP-9 mRNA expression may also play a role in preventing MBP degradation.

We can hypotize that the *in vivo* effect of IFN-β on CANP-2 and MMPs, produced by activated astrocytes or induced by pro-inflammatory cytokines, may contribute to reduce the amplification of inflammation and the subsequent axonal damage. Indeed, the degradation of MBP by MMPs and calpains would make the myelin sheath unstable and prone to lose its structural integrity and this, in turn, would make axons more susceptible to neurotoxic factors. The contribution of the intracellular enzyme CANP-2 to the degradation of MBP *in vivo* may be explained considering that in course of MS, activated astrocytes can undergo apoptosis [39], [40] and this would allow to CANP-2 to be released and come in contact with MBP produced by oligodendrocytes.

Studies in animal models of neuroinflammatory diseases have indicated that the expression of calpains increases with the severity of the disease, suggesting that the onset and severity of clinical symptoms could be related to this proteolytic activity [41]. In addition, since the astrocytic processes are localized at the level of blood-brain barrier, the inhibitory effect of IFN-β on calpains could prevent the further entry of inflammatory cells from peripheral blood into the CNS reducing demyelination and axonal injury.

Recent developments in TIMP research suggest that the activation of glial cells with pro-inflammatory cytokines and LPS results in an increased expression of TIMP-1, probably as a regulatory mechanism to control the activation of MMP-9 in inflammatory sites [37], [42]. Studies aimed at investigating the effect of IFN-β on TIMP levels have shown an increase in serum TIMP-1 following IFN-β treatment of patients [22]. Based on our knowledge, there are no studies establishing the effect of IFN-β on LPS-activated astrocytes. In our experimental system, the reduced expression of TIMP-1 and TIMP-2, following treatment with IFN-β may represent a compensatory mechanism to control the excessive production of TIMP, which often is related with the occurrence of fibrotic processes [43]. This result is consistent with the demonstration that antiretroviral drugs reduce TIMP-1 levels in LPS-activated astrocytes [33]. However, how the inhibition of MMP and TIMP-1 expression by IFN-β might affect *in vivo* the ratio MMP/TIMP remains to be established.

## Conclusions

In this study we provided evidence that the activation of rat astrocytes with LPS induces the activity and the expression of different types of MBP-cleaving proteolytic activities which were identified as calpain II (CANP-2) and gelatinases A (MMP-2) and B (MMP-9). These proteinases and their endogenous inhibitors were differently inhibited by IFN-β, which is particularly effective in inhibiting CANP-2.

These results support further the hypothesis that the neuroinflammatory damage during MS involves altered balance between multiple proteinases and their inhibitors and indicate that IFN-β is effective in regulating different enzymatic systems involved in MS pathogenesis. The inhibitory effect of IFN-β on the different proteolytic systems of MMP and calpains could represent a mechanism by which IFN-β may counteract demyelination and therefore decrease the development of new lesions in the course of MS.

## References

[1] Yong VW, Zabad RK, Agrawal S, Goncalves Dasilva A, Metz LM (2007) Elevation of matrix metalloproteinases (MMPs) in multiple sclerosis and impact of immunomodulators. J Neurol Sci 259: 79–84. 17382965 10.1016/j.jns.2006.11.021

[2] Shields DC, Schaecher KE, Saido TC, Banik NL (1999a) A putative mechanism of demyelination in multiple sclerosis by a proteolytic enzyme, calpain. Proc Natl Acad Sci U S A 96: 11486–11491. 10500203 10.1073/pnas.96.20.11486 PMC18060

[3] Gveric D, Hanemaaijer R, Newcombe J, van Lent NA, Sier CF, et al (2001) Plasminogen activators in multiple sclerosis lesions: implications for the inflammatory response and axonal damage. Brain 124: 1978–1988. 11571216 10.1093/brain/124.10.1978

[4] Hassen GW, Feliberti J, Kesner L, Stracher A, Mokhtarian F (2006) A novel calpain inhibitor for the treatment of acute experimental autoimmune encephalomyelitis. J Neuroimmunol 180: 135–146. 17007940 10.1016/j.jneuroim.2006.08.005

[5] Shields DC, Tyor WR, Deibler GE, Hogan EL, Banik NL (1998) Increased calpain expression in activated glial and inflammatory cells in experimental allergic encephalomyelitis. Proc Natl Acad Sci U S A 95: 5768–5772. 9576959 10.1073/pnas.95.10.5768 PMC20454

[6] Deshpande RV, Goust JM, Hogan EL, Banik NL (1995) Calpain secreted by activated human lymphoid cells degrades myelin. J Neurosci Res 42: 259–265. 8568927 10.1002/jnr.490420214

[7] Diaz-Sanchez M, Williams K, DeLuca GC, Esiri MM (2006) Protein co-expression with axonal injury in multiple sclerosis plaques. Acta Neuropathol 111: 289–299. 16547760 10.1007/s00401-006-0045-0

[8] Schaecher K, Rocchini A, Dinkins J, Matzelle DD, Banik NL (2002) Calpain expression and infiltration of activated T cells in experimental allergic encephalomyelitis over time: increased calpain activity begins with onset of disease. J Neuroimmunol 129: 1–9. 12161014 10.1016/s0165-5728(02)00142-x

[9] Shields DC, Banik NL (1999) Pathophysiological role of calpain in experimental demyelination. J Neurosci Res 55: 533–541. 10082076 10.1002/(SICI)1097-4547(19990301)55:5<533::AID-JNR1>3.0.CO;2-8

[10] Bartus RT, Elliott PJ, Hayward NJ, Dean RL, Harbeson S, et al (1995) Calpain as a novel target for treating acute neurodegenerative disorders. Neurol Res 17: 249–258. 7477738 10.1080/01616412.1995.11740322

[11] Rosenberg GA (2002) Matrix metalloproteinases in neuroinflammation. Glia 39: 279–291. 12203394 10.1002/glia.10108

[12] Opdenakker G, Nelissen I, Van Damme J (2003) Functional roles and therapeutic targeting of gelatinase B and chemokines in multiple sclerosis. Lancet Neurol 2: 747–756. 14636780 10.1016/s1474-4422(03)00587-8

[13] Steinman L, Gijbels K (1994) Gelatinase B producing cells in multiple sclerosis lesions. J Cell Biochem Suppl. 18D: 143.

[14] Gijbels K, Masure S, Carton H, Opdenakker G (1992) Gelatinase in the cerebrospinal fluid of patients with multiple sclerosis and other inflammatory neurological disorders. J Neuroimmunol 41: 29–34. 1334098 10.1016/0165-5728(92)90192-n

[15] Liuzzi GM, Trojano M, Fanelli M, Avolio C, Fasano A, et al (2002) Intrathecal synthesis of matrix metalloproteinase-9 in patients with multiple sclerosis: implication for pathogenesis. Mult Scler 8: 222–228. 12120694 10.1191/1352458502ms800oa

[16] Cuzner ML, Gveric D, Strand C, Loughlin AJ, Paemen L, et al (1996) The expression of tissue-type plasminogen activator, matrix metalloproteases and endogenous inhibitors in the central nervous system in multiple sclerosis: comparison of stages in lesion evolution. J Neuropathol Exp Neurol 55: 1194–1204. 8957442 10.1097/00005072-199612000-00002

[17] Proost P, Van Damme J, Opdenakker G (1993) Leukocyte gelatinase B cleavage releases encephalitogens from human myelin basic protein. Biochem Biophys Res Commun 192: 1175–1181. 7685161 10.1006/bbrc.1993.1540

[18] Chandler S, Coates R, Gearing A, Lury J, Wells G, et al (1995) Matrix metalloproteinases degrade myelin basic protein. Neurosci Lett 201: 223–226. 8786845 10.1016/0304-3940(95)12173-0

[19] Asahi M, Wang X, Mori T, Sumii T, Jung JC, et al (2001) Effects of matrix metalloproteinase-9 gene knock-out on the proteolysis of blood-brain barrier and white matter components after cerebral ischemia. J Neurosci 21: 7724–7732. 11567062 10.1523/JNEUROSCI.21-19-07724.2001 PMC6762894

[20] Trojano M, Avolio C, Liuzzi GM, Ruggieri M, Defazio G, et al (1999) Changes of serum sICAM-1 and MMP-9 induced by rIFNbeta-1b treatment in relapsing-remitting MS. Neurology 53: 1402–1408. 10534242 10.1212/wnl.53.7.1402

[21] Waubant E, Goodkin D, Bostrom A, Bacchetti P, Hietpas J, et al (2003) IFNbeta lowers MMP-9/TIMP-1 ratio, which predicts new enhancing lesions in patients with SPMS. Neurology 60: 52–57. 12525717 10.1212/wnl.60.1.52

[22] Gilli F, Bertolotto A, Sala A, Hoffmann F, Capobianco M, et al (2004) Neutralizing antibodies against IFN-beta in multiple sclerosis: antagonization of IFN-beta mediated suppression of MMPs. Brain 127: 259–268. 14607790 10.1093/brain/awh028

[23] Nakajima K, Hamanoue M, Shimojo M, Takei N, Kohsaka S (1989) Characterization of microglia isolated from a primary culture of embryonic rat brain by a simplieded method. Biomed Res 10: 411–423.

[24] McCarthy KD, de Vellis J (1980) Preparation of separate astroglial and oligodendroglial cell cultures from rat cerebral tissue. J Cell Biol 85: 890–902. 6248568 10.1083/jcb.85.3.890 PMC2111442

[25] Deibler GE, Martenson RE, Kies MW (1972) Large scale preparation of myelin basic protein from central nervous tissue of several mammalian species. Prep Biochem 2: 139–165. 4623901 10.1080/00327487208061467

[26] Croall DE, Moffett K, Hatch H (2002) Casein zymography of calpains using a 4-(2-hydroxyethyl)-1-piperazineethanesulfonic acid-imidazole buffer. Anal Biochem 304: 129–132. 11969198 10.1006/abio.2001.5606

[27] McLellan T (1982) Electrophoresis buffers for polyacrylamide gels at various pH. Anal Biochem 126: 94–99. 7181120 10.1016/0003-2697(82)90113-0

[28] Liuzzi GM, Latronico T, Fasano A, Carlone G, Riccio P (2004) Interferon-beta inhibits the expression of metalloproteinases in rat glial cell cultures: implications for multiple sclerosis pathogenesis and treatment. Mult Scler 10: 290–297. 15222694 10.1191/1352458504ms1016oa

[29] Rosenberg GA, Kornfeld M, Estrada E, Kelley RO, Liotta LA, et al (1992) TIMP-2 reduces proteolytic opening of blood–brain barrier by type IV collagenase. Brain Res 576: 203–207. 1381261 10.1016/0006-8993(92)90681-x

[30] Benveniste EN (1995) TNF-alpha- and IFN-gamma-mediated signal transduction pathways: effects on glial cell gene expression and function. Faseb J 9: 1577–1584. 8529837 10.1096/fasebj.9.15.8529837

[31] Leppert D, Waubant E, Galardy R, Bunnett N, Hauser SL (1995) T-cell gelatinases mediate basement membrane transmigration in vitro. J Immunol 154: 4379–4389. 7722295

[32] Liuzzi GM, Riccio P, Dal Canto MC (1995) Release of myelin basic protein-degrading proteolytic activity from microglia and macrophages after infection with Theiler's murine encephalomyelitis virus: comparison between susceptible and resistant mice. J Neuroimmunol 62: 91–102. 7499498 10.1016/0165-5728(95)00110-n

[33] Liuzzi GM, Mastroianni CM, Latronico T, Mengoni F, Fasano A, et al (2004b) Anti-HIV drugs decrease the expression of matrix metalloproteinases in astrocytes and microglia. Brain 127: 398–407. 14662518 10.1093/brain/awh049

[34] Opdenakker G, Van Damme J (2011) Probing cytokines, chemokines and matrix metalloproteinases towards better immunotherapies of multiple sclerosis. Cytokine Growth Factor Rev 22: 359–65. 22119009 10.1016/j.cytogfr.2011.11.005

[35] Guyton MK, Brahmachari S, Das A, Samantaray S, Inoue J, et al (2009) Inhibition of calpain attenuates encephalitogenicity of MBP-specific T cells. J Neurochem 110: 1895–1907. 19627443 10.1111/j.1471-4159.2009.06287.x PMC2748265

[36] Guyton MK, Das A, Samantaray S, Wallace GC 4th, Butler JT, et al (2010) Calpeptin attenuated inflammation, cell death, and axonal damage in animal model of multiple sclerosis. J Neurosci Res 88: 2398–2408. 20623621 10.1002/jnr.22408 PMC3164817

[37] Gomez DE, Alonso DF, Yoshiji H, Thorgeirsson UP (1997) Tissue inhibitors of metalloproteinases: structure, regulation and biological functions. Eur J Cell Biol 74: 111–122. 9352216

[38] Bartholomé EJ, Van Aelst I, Koyen E, Kiss R, Willems F, et al (2001) Human monocyte-derived dendritic cells produce bioactive gelatinase B: inhibition by IFN-beta. J Interferon Cytokine Res. 21: 495–501. 10.1089/10799900152434367 11506743

[39] Benjelloun N, Ménard A, Charriaut-Marlangue C, Mokhtari K, Perron H, et al (1998) Case report: DNA fragmentation in glial cells in a cerebral biopsy from a multiple sclerosis patient. Cell Mol Biol. 44: 579–83.). 9678892

[40] Dowling P, Shang G, Raval S, Menonna J, Cook S, et al (1996) Involvement of the CD95 (APO-1/Fas) receptor/ligand system in multiple sclerosis brain. J Exp Med. 184: 1513–8. 10.1084/jem.184.4.1513 PMC2192814 8879222

[41] Guyton MK, Wingrave JM, Yallapragada AV, Wilford GG, Sribnick EA, et al (2005) Upregulation of calpain correlates with increased neurodegeneration in acute experimental auto-immune encephalomyelitis. J Neurosci Res 81: 53–61. 15952172 10.1002/jnr.20470

[42] Suryadevara R, Holter S, Borgmann K, Persidsky R, Labenz-Zink C, et al (2003) Regulation of tissue inhibitor of metalloproteinase-1 by astrocytes: links to HIV-1 dementia. Glia 44: 47–56. 12951656 10.1002/glia.10266 PMC3820378

[43] Mastroianni CM, Liuzzi GM, D'Ettorre G, Lichtner M, Forcina G, et al (2002) Matrix metalloproteinase-9 and tissue inhibitors of matrix metalloproteinase-1 in plasma of patients co-infected with HCV and HIV. HIV Clin Trials 3: 310–315. 12187505 10.1310/U9LJ-MFF9-ARE1-257H

